# Retrospective Longitudinal Study on Changes in Atmospheric Pressure as a Predisposing Factor for Odontogenic Abscess Formation

**DOI:** 10.3390/dj11020042

**Published:** 2023-02-08

**Authors:** Marko Tarle, Arijan Zubović, Boris Kos, Marina Raguž, Ivica Lukšić

**Affiliations:** 1 Department of Maxillofacial Surgery, Dubrava University Hospital, Gojko Šušak Avenue 6, 10000 Zagreb, Croatia; 2 School of Dental Medicine, University of Zagreb, Gundulićeva 5, 10000 Zagreb, Croatia; 3 Department of Maxillofacial Surgery, Clinical Hospital Center Rijeka, Krešimirova 42, 51000 Rijeka, Croatia; 4 The Faculty of Medicine, University of Rijeka, Braće Branchetta 20/1, 51000 Rijeka, Croatia; 5 Department of Neurosurgery, Dubrava University Hospital, Gojko Šušak Avenue 6, 10000 Zagreb, Croatia; 6 School of Medicine, Catholic University of Croatia, Ilica 242, 10000 Zagreb, Croatia; 7 School of Medicine, University of Zagreb, Šalata 3, 10000 Zagreb, Croatia

**Keywords:** odontogenic abscess, atmospheric pressure, weather changes, antibiotics, maxillofacial infections

## Abstract

In our retrospective longitudinal study based on the data from 292 patients, we wanted to investigate whether there was an association between weather conditions and the occurrence of odontogenic abscesses (OA) requiring hospitalization. In the adult group (249 patients), the incidence of severe OA was highest in winter (32.9%) during January (11.6%), with the most common localizations being the perimandibular (35.7%) and submandibular (23.3%) regions. We found that changes in mean daily atmospheric pressure five days before hospitalization showed a positive association with the occurrence of OA, especially pressure variations greater than 12 hPa. Atmospheric pressure changes two and five days before hospitalization were also found to be moderate predictors of complications during treatment. Antibiogram analysis revealed resistance of streptococci to clindamycin in 26.3%. In the pediatric group, OA were also most frequent in winter (30.2%), and the perimandibular region (37.2%) and the canine fossa (20.9%) were the most frequent abscess localizations, while an association with meteorological parameters was not demonstrated. Clinical experience teaches us that weather change influences the occurrence of severe OA requiring hospitalization, which we confirmed in this research. To our knowledge, our study is the first to provide a threshold and precise time frame for atmospheric pressure changes.

## 1. Introduction

Odontogenic abscesses (OA) are one of the most common emergencies in oral and maxillofacial surgery and are usually endogenous aerobic/anaerobic mixed infections caused by facultative microorganisms from the oral cavity [1]. The most common causes are infections after tooth extraction, teeth with apical periodontitis, and retained or impacted teeth with pericoronitis. The submandibular and perimandibular spaces are the most common sites of OA, accounting for 42.5% and 18.5%, respectively [2]. The requirement for intravenous antibiotic treatment, surgical incision and drainage under local or general anesthesia, and other factors in many of these instances necessitate hospitalization [3,4,5]. Oral bacteria and most commensal microbiota are adapted to their environment and show the highest growth rates at 37 °C, while they decrease at lower and higher temperatures [6]. Therefore, it is still a common belief among dentists and oral surgeons that the occurrence of OA may be related to a meteorological season or weather conditions such as temperature or atmospheric pressure. In 1981, Harlfinger et al. were able to find a relationship between changes from anticyclonic to cyclonic conditions and frequency of OA [7]. In 2015, Seemann et al. reported that low atmospheric pressure increased the number of patients with OA [8]. On the other hand, in the largest study, which included 2111 patients with OA, no correlation was found between a surrogate parameter of temperature and the occurrence of abscesses, while in the study by Meningaud et al., no correlation was found between average atmospheric pressure or temperature and oral abscesses in 301 patients over a one-year period [9,10]. There are few publications that establish a relationship between meteorological parameters and the occurrence of OA. Whether the occurrence of OA is influenced by outside weather conditions or other environmental factors remains largely unanswered [8,11]. The aim of this study was to investigate the influence of meteorological parameters on the occurrence of OA and to compare the results with clinical data in both adult and pediatric patients.

## 2. Materials and Methods

For each patient in our study group, the geographic location above sea level and the time of onset of symptoms were recorded. Weather data and indicators were obtained from the Croatian Meteorological and Hydrological Service (https://meteo.hr/, accessed on 18 December 2021). The following meteorological parameters were recorded for each patient on the day of hospitalization and two, five, and seven days before hospitalization: daily maximum, minimum, and mean ambient temperature (°C), daily mean relative humidity (%), daily mean atmospheric pressure (hPa), and precipitation. Changes in meteorological parameters were calculated as differences between the day of and two, five, and seven days before hospitalization. All data were checked for accuracy by two independent researchers.

A medical history was obtained from all patients, and a standardized clinical examination was performed before the procedure. The exact localization of the OA was taken from the surgical report. Patients with insufficient/contradictory data were not included. Blood analyses, including white blood cell (WBC) count and C-reactive protein (CRP), and dental panoramic radiographs or computed tomography scans (CT) were performed in all patients. In most patients in whom surgical incision was made, a wound swab was taken and microbiological analysis was performed. Intravenous antibiotics were administered to all patients. Postoperative treatment included local cooling and physical rest.

Drains were checked daily and trimmed every day. Trismus was treated with mouth-opening exercises. If necessary, a check-up CT and extraction of the causative tooth were performed.

In addition, the following parameters were analyzed: age, sex, seasonal frequency, causative tooth, therapy used, previous dental intervention, antibiogram, antibiotics used, antibiotic prophylaxis, pathogen groups, comorbidities, complications, localization of abscess, inflammatory parameters (WBC, CRP), days from symptom onset to hospitalization, days from dental procedure to hospitalization, and hospitalization days. Data analysis was performed with MedCalc Statistical Software version 12.5.0 (MedCalc Software, Ostend, Belgium; https://www.medcalc.org, accessed on 14 August 2022). The distribution was assessed with the Kolmogorov–Smirnov test. The chi-square test was used for qualitative variables. Associations between meteorological parameters and other various patient data were assessed with the Pearson correlation coefficient or Spearman’s rank correlation. The potential value for predicting the occurrence of complications was assessed using receiver-operating characteristic (ROC) curve analysis. Statistical significance was set at *p* < 0.05.

## 3. Results

Between 1 January 2015 and 1 June 2021, a total of 292 consecutive patients (168 males and 124 females) were enrolled in the study. Admitted patients were initially divided into two groups, the pediatric group and the adult group, according to dentition. The pediatric group included 43 patients, 27 males and 16 females, while the adult group included 249 patients, 141 males and 108 females. The mean age of the included adult patients was 38.38 ± 11.28 years, with a mean age of 38.29 ± 15.83 in male patients and 38.26 ± 15.81 in female patients, while the mean age of the included pediatric patients was 6.47 ± 2.96 years, with a mean age of 6.41 ± 2.94 in male patients and 6.56 ± 3.10 in female patients. In the adult group, we first present the data by season (spring, summer, fall, winter); the seasonal incidence peaked in winter at 32.90% and showed a low of 24.90% in spring, 22.5% in fall, and 19.7% in summer (*p* = 0.03). Analysis of incidence by month of hospitalization showed a peak in January, 11.6%, December, 10%, and February and May, 9.6% (*p* = 0.06).

Daily mean temperature (r = 0.03, *p* = 0.58), relative humidity (r = 0.17, *p* = 0.24), atmospheric pressure (r = 0.15, *p* = 0.71), and precipitation (r = 0.01, *p* = 0.98) showed no association with the incidence of OA at the time of hospital admission (Table 1). Changes in meteorological parameters were calculated as differences between the day and two, five, and seven days before hospitalization. Changes in daily mean barometric pressure five days prior to hospitalization showed a positive association with the occurrence of OA (r = 0.48, *p* = 0.05) (Table 1). Interestingly, changes greater than 12 hPa five days prior to hospitalization showed a strong positive association with the occurrence of OA (r = 0.52, *p* = 0.05).

Regarding other parameters, we observed interesting results. Although antibiogram was not obtained in 64.3% of patients, interestingly, in 26.3% of patients, clindamycin resistance was observed. Nevertheless, in our cohort, a combination of co-amoxiclav and metronidazole was used in 79.5% of patients (*p* < 0.00001), while clindamycin was used in only 7.2% of cases. In addition, antibiotic prophylaxis was used in 50.8% of the included patients. However, we must mention that due to the retrospective study design, this information is unknown in 30% of patients. For the same reason, the pathogens were unknown in almost 50% of the included patients. However, it should be mentioned that streptococci were observed in 28.9% of cases and staphylococci in 4.4% of cases.

**Figure 1:**
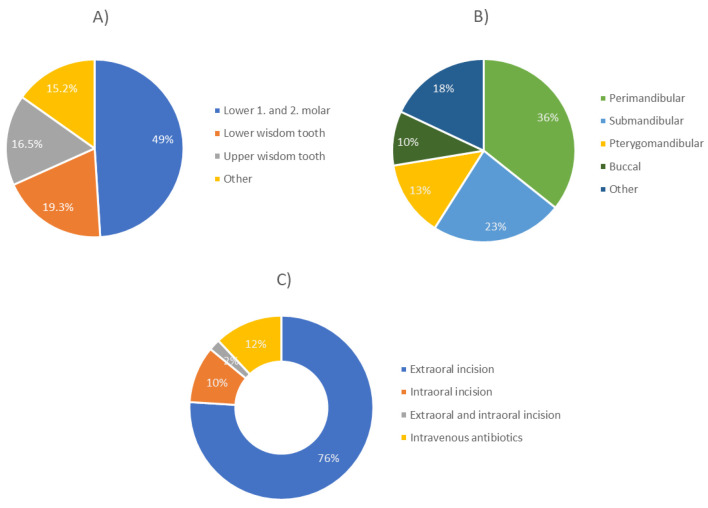
Distribution regarding causative tooth (**A**), abscess localization (**B**), and treatment modality (**C**) in adult patients with severe odontogenic abscess.

Before the occurrence of OA, a tooth procedure was performed in 70.7% of patients (*p* < 0.0001). In 49% of patients, the causative teeth were lower first and second molars, followed by lower (19.3%) and upper wisdom tooth (16.5%). Regarding the localization of the abscess, perimandibular (35.7%) and submandibular (23.3%) abscesses were observed most frequently, followed by pterygomandibular (13.4%) and buccal abscesses (9.6%). Extraoral incision was performed in most patients (75.9%), followed by intraoral incision (10%) and a combination of both methods (2%). Twelve % of patients were treated with antibiotics only (Figure 1). No complications were observed in most patients (94%). Re-incision was performed in 5.2% of patients, while only one case of necrotizing fasciitis-mediastinitis and one case of osteo-myelitis were observed (0.4%). Most patients had no comorbidities (75.9%), while others had various diseases such as arterial hypertension or diabetes.

Association between different clinical parameters (Table 2); a significant association was found between the occurrence of complications and hospitalization days (*p* = 0.83, r = 0.01, R^2^ = 0.0001) and the increase in CRP levels (*p* = 0.05, r = 0.12, R^2^ = 0.015), WBC count increase (*p* = 0.62, r = 0.03, R^2^ = 0.0001), antibiotics (*p* = 0.31, r = 0.06, R^2^ = 0.003), and comorbidities (*p* = 0.154, r = 0.08, R^2^ = 0.007) showed no association. In addition, hospital days showed a significant association with the increase in CRP level (*p* = 0.01, r = 0.25, R^2^ = 0.063), in contrast to the increase in WBC count (*p* = 0.85, r = 0.01, R^2^ = 0.0001) and antibiotics (*p* = 0.07, r = 0.11, R^2^ = 0.01).

**Figure 2:**
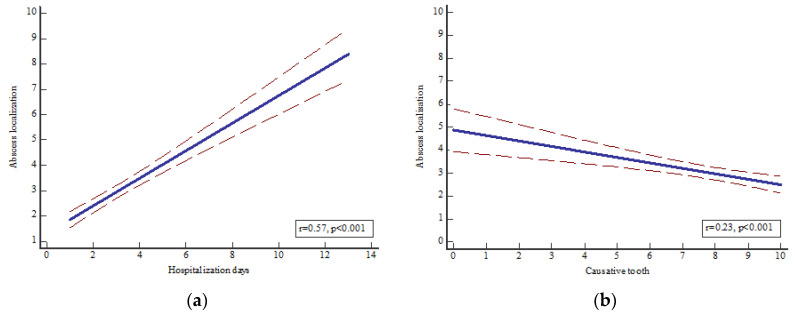
Significant association between (**a**) OA localization and hospitalization days (*p* < 0.001, r = 0.57, R^2^ = 0.33) and (**b**) causative tooth (*p* < 0.0001, r = 0.23, R^2^ = 0.053). A 95% confidence interval has been presented. Legend: abscess localization: 1—perimandibular, 2—submandibular, 3—pterygomandibular, 4—buccal, 5—submucosal, 6—fossa canina, 7—submental, 8—sublingual, 9—peri/submandibular + pterygomandibular, 10—fasciitis; tooth: 1—upper incisors, 2—upper canines, 3—upper premolars, 4—upper molars, 5—upper wisdom teeth, 6—lower incisors, 7—lower canines, 8—lower premolars, 9—lower molars, 10—lower wisdom teeth.

A significant association was observed between the localization of the OA and the causative tooth (*p* < 0.0001, r = 0.23, R^2^ = 0.053) and hospitalization days (*p* < 0.001, r = 0.57, R^2^ = 0.33) (Figure 2), as well as between the causative tooth and hospitalization days (*p* < 0.0001, r = 0.41, R^2^ = 0.167).

**Figure 3:**
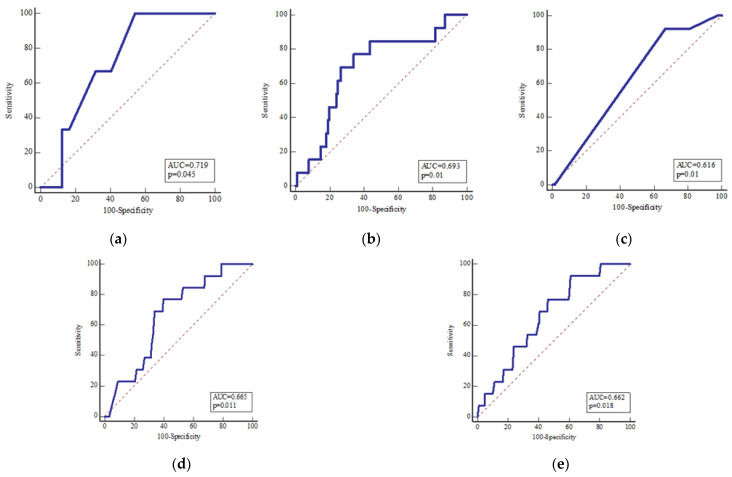
The value of meteorological and clinical parameters in predicting potential complications, ROC analysis: (**a**) number of days from intervention to hospitalization (area under curve, AUC = 0.72; *p* = 0.04, sensitivity, SE = 100%, specificity, SP = 45.6%), (**b**) CRP levels increase (AUC = 0.69; *p* = 0.01, SE = 76.9%, SP = 66.1%), (**c**) type of therapy of OA (AUC = 0.62; *p* = 0.01, SE = 92.3%, SP = 33.2%), (**d**) change of atmospheric pressure two days prior to hospitalization (AUC = 0.66; *p* = 0.01, SE = 76.9%, SP = 60.3%), and (**e**) change of atmospheric pressure five days prior to hospitalization (AUC = 0.66; *p* = 0.01, SE = 92.3%, SP = 39.0%) present moderate to strong indicators for potential complications.

To determine the value of meteorological and clinical parameters in predicting potential complications, an ROC analysis was performed. The number of days between intervention and hospitalization are moderate indicators of possible complications (area under the curve, AUC = 0.72; *p* = 0.04, sensitivity, SE = 100%, specificity, SP = 45.6%), in contrast to days elapsed since symptom onset (AUC = 0.58; *p* = 0.34, SE = 58.23%, SP = 64.7%), OA localization (AUC = 0.55; *p* = 0.48, SE = 76.9%, SP = 36.5%), or hospitalization days (AUC = 0.51; *p* = 0.84, SE = 0.0%, SP = 78.7%). In addition, the increase in CRP level (AUC = 0.69; *p* = 0.01, SE = 76.9%, SP = 66.1%) and type of therapy for OA (AUC = 0.62; *p* = 0.01, SE = 92.3%, SP = 33.2%) represent moderate to strong indicators of potential complications. Interestingly, the change in atmospheric pressure two (AUC = 0.66; *p* = 0.01, SE = 76.9%, SP = 60.3%) and five (AUC = 0.66; *p* = 0.01, SE = 92.3%, SP = 39.0%) days before hospitalization is also a moderate indicator of possible complications (Figure 3).

**Figure 4:**
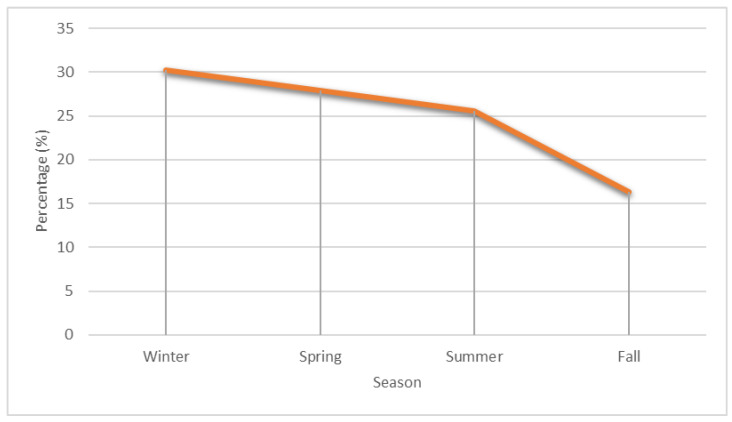
Seasonal incidence of odontogenic abscesses in the pediatric population.

In the pediatric group, seasonal incidence peaked in winter at 30.2% and showed a low point in spring at 27.9%, summer at 25.6%, and fall at 16.3% (*p* = 0.06) (Figure 4). Analysis of incidence by month of hospitalization showed a peak in January, May, and August, 14% (*p* = 0.08). Daily mean temperature (r = 0.09, *p* = 0.73), relative humidity (r = 0.07, *p* = 0.66), atmospheric pressure (r = 0.22, *p* = 0.07), and precipitation (r = 0.13, *p* = 0.81), as well as changes in meteorological parameters two, five, and seven days before the onset of symptoms showed no association with the incidence of OA at the time of symptom onset in the pediatric group. No antibiogram was obtained in almost 80% of pediatric patients, and clindamycin resistance was detected in 4.7% of patients. Co-amoxiclav was used in 48.8% of patients, while co-amoxiclav and metronidazole was used in 34.9% of patients.

**Figure 5:**
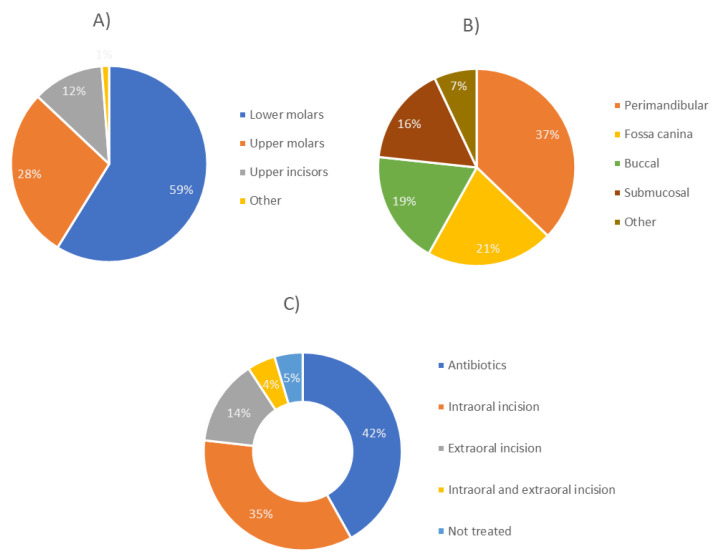
Distribution regarding causative tooth (**A**), abscess localization (**B**), and treatment modality (**C**) in pediatric patients with odontogenic abscess.

Antibiotic prophylaxis was not used in 61.3% of the included patients. In 76.7% of the included patients, the pathogens were unknown. Nevertheless, it should be mentioned that streptococci were observed in 9.3% of cases and staphylococci in 2.3% of cases. Before the occurrence of OA, previous tooth procedure was performed in 72.0% of patients (*p* < 0.001). In 58.1% of patients, the causative teeth were lower molars, followed by upper molars (27.9%) and upper incisors (11.6%) (Figure 5). As for the localization of the OA, perimandibular (37.2%) and fossa canina (20.9%) were observed most frequently, followed by buccal (18.6%) and submucosal (16.3%) (Figure 5). 

The majority of patients were treated with antibiotics only (41.9%), followed by intraoral incision (34.9%), extraoral incision (14%), and a combination of both methods (4.6%); 4.6% of patients were not treated (Figure 5). Most patients were without comorbidities (93%), and no complications were observed in the pediatric group.

**Figure 6:**
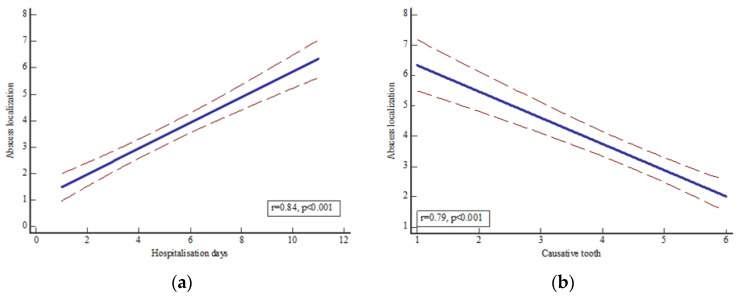
Significant association between (**a**) OA localization and hospitalization days (*p* < 0.0001, r = 0.84, R^2^ = 0.71) and (**b**) abscess localization and causative tooth (*p* < 0.0001, r = 0.79, R^2^ = 0.63). Legend: abscess localization: 1—perimandibular, 2–submandibular, 3—pterygomandibular, 4—buccal, 5—submucosal, 6—fossa canina, 7—submental, 8—sublingual; tooth: 1—upper incisors, 2—upper canines, 3—upper molars, 4—lower incisors, 5—lower canines, 6—lower molars.

A significant association was observed between OA localization and hospitalization days (*p* < 0.0001, r = 0.84, R^2^ = 0.71) and causative tooth (*p* < 0.0001, r = 0.79, R^2^ = 0.63) (Figure 6). In addition, hospitalization days showed no association with increase in CRP level (*p* = 0.93, r = 0.01, R^2^ = 0.0001), increase in WBC count (*p* = 0.37, r = 0.14, R^2^ = 0.02), or antibiotics (*p* = 0.57, r = 0.09, R^2^ = 0.007). A moderate association was observed between the month of hospital admission and the localization of OA (*p* = 0.002, r = 0.46, R^2^ = 0.22), while the season of admission, changes in meteorological parameters, and the previously mentioned clinical parameters were not observed. 

## 4. Discussion

We divided our patients into adult and pediatric groups based on differences in dentition. The American Journal of Dental Association reports that most children have their permanent teeth by the age of 13, so we set our cutoff at 13 years [12]. In terms of gender distribution, our study is comparable to that of Seemaan et al., with a slightly higher representation of the male gender [8]. This could be due to better dental hygiene and toothbrushing practices in females, as shown by Mamai-Homata et al. [13]. The age distribution in our adult group is comparable to that in the study by Seeman et al. (39.0 ± 21.9) and in the study by Spalthoff et al. (40.1) [8,11]. One might expect the prevalence to be higher in the elderly population because they are generally more susceptible to infection. However, there is also a higher prevalence of edentulousness among individuals older than 45 years, as shown by Jukić- Krmek et al. in their study of the population of the Croatian town of Knin [14]. Therefore, one cannot expect a high incidence of OA in this age group.

In our study, there was no correlation between the incidence of OA formation at the time of hospitalization and daily average temperature, relative humidity, atmospheric pressure, or precipitation. These results are consistent with the studies of Spalthoff et al., who found no relationship between climatic parameters and intraoral or extraoral abscess incidence [11]. Seemann et al. also discovered no relationship between an intraoral incision and the average daily temperature [8]. According to studies by Nissen and Schmidsedar and Harlfinger and Graup, there was no correlation between the incidence of abscesses and the external temperature [7,15]. Assessing the effect of daily average temperature on the incidence of OA can be difficult because of the temperature fluctuations to which people are exposed throughout the day. As for the effects of temperature fluctuations on the pathophysiology of OA development, further studies should be considered. According to the study by Seemann et al., days with low atmospheric pressure were associated with a significant increase in the incidence of abscesses requiring intraoral incision [8]. 

Since we emphasize the importance of variations in atmospheric pressure, our study is consistent with the one mentioned earlier. In addition, it is consistent with the studies of Harlfinger and Graup, who found a relationship between an increase in abscess formation and anticyclonic-to-cyclonic shifts [7]. To our knowledge, our study is the first to provide a threshold for atmospheric pressure changes and to provide an accurate time frame for these changes. Our study complements the study by Nissen and Schmidsedar, who demonstrated an association between low barometric pressure and an increase in the incidence of OA formation [15]. Barodontalgia, the phenomenon of toothache triggered by a change in atmospheric pressure, could serve as an explanation [8,16,17]. Moreover, how atmospheric pressure affects the actual pain sensation has been studied [18]. Therefore, we hypothesize that a higher likelihood of hospitalization for more severe pain could explain the higher frequency of hospitalizations due to OA. It is also plausible to explain how variations in atmospheric pressure affect the development of OA by decreasing oxygen saturation. According to Dohmen L.M. et al., a decrease in atmospheric pressure of 1 hPa causes a decrease in oxygen saturation by 0.006 [19]. According to a study by Gupta et al., microbes are less sensitive to most of the regularly used antibiotics when oxygen levels are lower [19]. They successfully demonstrated this in staphylococcus aureus, a common isolate in our study. 

Further studies are needed to determine the connection between the exact decrease in blood oxygen levels and the susceptibility to pathogens observed in OA. As shown by Hajdamowicz et al., tissue hypoxia affects both pathogens and neutrophils and leads to abscess formation [20]. 

Their research provided an explanation of how the hypoxic tissue environment affects neutrophils and pathogens in a way that enhances intracellular persistence of infections, which together lead to abscess formation. OAs are characterized as osmotic systems that can be affected by increasing pressure in the environment. Therefore, increased tissue oxygen levels may inhibit abscess development [21]. Further research is needed to determine how tissue oxygen levels are related to the local tissue microenvironment in the craniofacial area. As far as we know, we were the first to report changes in atmospheric pressure as an indication of potential complications. This strengthens our argument that changes in atmospheric pressure play a pathophysiologic role in the development of OA. Regarding seasonality, The Department of Maxillofacial Surgery at Dubrava University Hospital provides 24 h emergency outpatient services throughout the year, so this should not affect seasonality. According to Fares et al., environmental factors, including indoor activity and vitamin D intake, have a general influence on seasonal variation in infectious diseases [22]. In addition, melatonin levels vary between summer and winter days due to sun exposure, and melatonin has been cited in research as an important immune modulator [22,23,24].

Most OA are mixed infections with facultative pathogenic microorganisms. These usually include aerobic streptococci and Gram-negative anaerobic bacteria [25]. In our study, streptococci were observed in 28.90% of cases and staphylococci in 4.4% of cases. In the study of Doll et al., streptococcus (27%), fusobacterium species (19%), prevotella species (16%), and bacteroides species (14%) were the most frequently isolated microorganisms. In another study, the most frequently found facultative anaerobes belonged to the viridans group streptococci and the anginosus group streptococci, while some studies indicated that staphylococci may be a more common colonizer of oral tissues than previously thought [26,27]. Interestingly, it has been reported that staphylococcus aureus is more common in severe OA in children [28,29]. In their study, De Jonge et al. found that perioperative antibiotic prophylaxis is best when administered in a time frame of 120 min before surgery, while Cohen et al. showed that the use of antibiotic prophylaxis is not associated with postoperative antibiotic-resistant infections [17,30]. Most of the bacterial species that cause endodontic infections, including abscesses, are sensitive to penicillin [31,32,33]. When empirical antibiotics are again required, amoxicillin remains the antibiotic of first choice [34]. 

When antimicrobial results indicate a high prevalence of resistance to amoxicillin, amoxicillin in combination with clavulanic acid and metronidazole should be considered as an alternative [35,36]. In our study, 26.3% of patients showed resistance to clindamycin. Clindamycin has been shown to be a good substitute for the treatment of acute OA, has potent antimicrobial activity against oral anaerobes, and is an effective alternative in patients allergic to penicillin [34,37]. As in our study, increased resistance to clindamycin has been reported in odontogenic infections. Shakya et al. found clindamycin resistance up to 15% [1,38]. However, compared with penicillin, clindamycin may also have side effects and carry the risk of developing pseudomembranous colitis [39]. 

In contrast to a study by Mair et al. which found that 6.0% of 184 patients hospitalized with dental abscesses had previously undergone root canal treatment or tooth extraction, the adult group showed a significantly higher correlation between previous dental treatment and the frequency of OA formation [40]. We explain such a discrepancy due to socioeconomic and organizational differences in the health care system. 

Regarding the frequency of causative teeth, our experience is consistent with the relevant literature [40,41]. According to other studies, infections occur more frequently in the submandibular region [40,41,42,43,44]. This discrepancy may be explained by the subjectivity of clinical and radiological findings.

It could also be argued that the timing of presentation could also contribute to the differences described above, as the perimandibular space is a continuation of the infection of the submandibular space. 

In addition, we demonstrated that the localization of the OA influences the number of days spent in the hospital. According to the study by Peters et al., the location of the infection predicts how long patients stay in the hospital [45,46]. On the other hand, Bowe et al. claimed that the number of affected fascial spaces is a predictor of the length of hospital stay [45]. Since the fascial spaces in the head and neck are connected, an illness in one place typically precedes an infection in another. It makes sense that a deeper fascial space infection would result in a more severe infection. Regarding the treatment modality, the results of our study agreed with those of Bowe et al. [45]. The need for a second surgical procedure is comparable to the study of Flynn et al. In their study, 8.0% of patients required re-incision, whereas in our study this was the case in 5.2% of patients [41]. Most of our patients (74.9%) had no underlying comorbidities. According to another similar study, 8.0% of patients had a disease that affected their immune system [41]. Moreover, there was no correlation between concomitant diseases and the occurrence of complications in our study. In contrast to our results, Peters et al. have shown that underlying disease is the best predictor of the occurrence of complications.

In contrast to our results, Peters et al. have shown that underlying disease is the best predictor of the length of hospital stay [45,46]. We contend that because oral hygiene plays the largest role in dental infection, underlying comorbidities have a smaller impact. CRP, in contrast to WBC count, is significantly related to the occurrence of complications and hospital days, and is a strong indicator of potential complications in the analysis of ROC. This is consistent with the relevant literature [4,42,47]. Other authors point out that WBC levels on admission may also predict longer length of hospital stay [40]. CRP levels have been strongly associated with the severity of odontogenic infections in the recent literature [48]. Erythrocyte sedimentation rate (ESR) and WBC count are extensively studied indicators of inflammation, but without the sensitivity and temporal accuracy offered by CRP [4,49,50,51].

Since there is more time for infection to grow, we contend that the number of days between intervention and hospitalization is a potential signal of potential complications. The perceived timing of symptom onset is too variable to be a predictor for potential complications. Since the type of therapy relies on the severity of the infection, it makes sense that more aggressive therapeutic modalities are associated with more serious diseases.

As in previous studies of OA in the pediatric population, there was a slight predominance of the male sex in our pediatric group [25,52]. In other studies, the distribution is even [53,54]. The incidence peaks in winter and is comparable to that of an adult group as previously explained. As in comparable studies, penicillin was the drug of choice for the pediatric population [52]. The number of children receiving antibiotic prophylaxis at the time of presentation is comparable to other studies [55]. In our study, streptococcus was the most common pathogen, which is consistent with the literature, and molars were the most common source of infection found, which is consistent with previous studies [25,53]. In this regard, our study is consistent with the localization of abscesses in other studies. Fossa canina, submucosal, and perimandibular abscesses are the most common according to a similar study [25]. 

Antibiotics were used exclusively to treat most patients. Similar studies have found that although children respond better to antibiotic therapy than adults, the targeted use of antibiotics in the pediatric group can postpone procedures [53]. There was a significant association between the causative tooth, the localization of the abscess, and the number of days spent in the hospital. This is in line with similar research by Doll et al. [25].

It is known that the causative tooth determines the severity of clinical presentation and the spread of infection. In our pediatric cohort, there was no association between climatic variables and the frequency of OA requiring hospitalization. 

The reason for this result is the small number of patients as well as the susceptibility of this group, so we hospitalized children with a less severe OA, who often required only intravenous antibiotic treatment with extraction of the causative tooth, without the need for an extraoral incision.

## 5. Conclusions

Our research found that changes in atmospheric pressure had an impact on the frequency of odontogenic abscesses requiring hospitalization. To the best of our knowledge, our study is the first to provide a threshold and precise time frame for atmospheric pressure changes. A change in atmospheric pressure greater than 12 hPa five days before hospitalization is associated with a higher number of hospitalizations, whereas greater pressure changes two and five days before hospitalization are moderate predictors of complications. Due to the high resistance of streptococci to clindamycin, it is not suitable for the treatment of OA; in particular, it should not be the antibiotic of first choice in penicillin-allergic patients.

## Figures and Tables

**Table 1:** Association of changes in meteorological parameters (mean temperature, relative humidity, atmospheric pressure, precipitation) and the occurrence of OA on the day of hospitalization and two, five, and seven days before hospitalization.

Meteorological Parameters	Hospital Admission	Two Days Prior to Hospitalization	Five Days Prior to Hospitalization	Seven Days Prior to Hospitalization
Δ mean temperature	r = 0.03, *p* = 0.58	r = 0.12, *p* = 0.78	r = 0.01, *p* = 0.86	r = 0.00, *p* = 0.88
Δ relative humidity	r = 0.17, *p* = 0.24	r = 0.01, *p* = 0.97	r = 0.01, *p* = 0.90	r = 0.01, *p* = 0.14
Δ atmospheric pressure	r = 0.23, *p* = 0.07	r = 0.24, *p* = 0.07	r = 0.48, *p* = 0.05	r = 0.19, *p* = 0.11
Δ precipitation	r = 0.01, *p* = 0.98	r = 0.11, *p* = 0.39	r = 0.02, *p* = 0.62	r = 0.02, *p* = 0.44

**Table 2:** Association between analyzed clinical parameters.

	Tooth	Intervention	Location	Prophylaxis	Complications	Hospital Days	Leukocyte	CRP	Antibiotics	Comorbidities	Type of Intervention
tooth		*p* = 0.823, r = 0.01, R^2^ = 0.0001	*p* < 0.0001, r = 0.23, R^2^= 0.053	*p* = 0.47, r = 0.04 R^2^ = 0.001	*p* = 0.989, r = 0.001, R^2^ = 0.0001	*p* < 0.0001, r = 0.41, R^2^= 0.167	*p* = 0.765, r = 0.02, R^2^ = 0.0003	*p* = 0.072, r = 0.11, R^2^ = 0.01	*p* = 0.490, r = 0.04, R^2^ = 0.001	*p* = 0.233, r = 0.07, R^2^ = 0.004	*p* = 0.886, r = 0.01, R^2^ = 0.0001
intervention	*p* = 0.823, r = 0.01, R^2^ = 0.0001		*p* = 0.317, r = 0.06, R^2^ = 0.003	*p* < 0.0001, r = 0.39, R^2^= 0.152	*p* = 0.916, r = 0.01, R^2^ = 0.0001	*p* = 0.473, r = 0.04, R^2^ = 0.001	*p* = 0.337, r = 0.06, R^2^ = 0.003	*p* = 0.408, r = 0.05, R^2^ = 0.002	*p* = 0.272, r = 0.06, R^2^ = 0.004	*p* = 0.297, r = 0.06, R^2^ = 0.003	*p* < 0.0001, r = 0.81, R^2^= 0.657
location	*p* < 0.0001, r = 0.23, R^2^= 0.053	*p* = 0.317, r = 0.06, R^2^ = 0.003		*p* = 0.852, r = 0.01, R^2^ = 0.0001	*p* = 0.331, r = 0.06, R^2^ = 0.003	*p* = 0.01, r = 0.25, R^2^ = 0.063	*p* = 0.887, r = 0.01, R^2^ = 0.00007	*p* = 0.014, r = 0.14, R^2^ = 0.02	*p* = 0.151, r = 0.08, R^2^ = 0.007	*p* = 0.441, r = 0.05, R^2^ = 0.002	*p* = 0.554, r = 0.05, R^2^ = 0.002
prophylaxis	*p* = 0.47, r = 0.04 R^2^ = 0.001	*p* < 0.0001, r = 0.39, R^2^= 0.152	*p* = 0.852, r = 0.01, R^2^ = 0.0001		*p* = 0.575, r = 0.03, R^2^ = 0.001	*p* = 0.529, r = 0.04, R^2^ = 0.001	*p* = 0.500, r = 0.04, R^2^ = 0.001	*p* = 0.940, r = 0.00, R^2^ = 0.00001	*p* < 0.001, r = 0.21, R^2^ = 0.004	*p* = 0.597, r = 0.03, R^2^ = 0.0009	*p* = 0.004, r = 0.22, R^2^ = 0.051
complications	*p* = 0.989, r = 0.001, R^2^ = 0.0001	*p* = 0.916, r = 0.01, R^2^ = 0.0001	*p* = 0.331, r = 0.06, R^2^ = 0.003	*p* = 0.575, r = 0.03, R^2^ = 0.001		*p* = 0.83, r = 0.01, R^2^ = 0.0001	*p* = 0.62, r = 0.03, R^2^ = 0.0001	*p* = 0.05, r = 0.12, R^2^ = 0.015	*p* = 0.314, r = 0.06, R^2^ = 0.003	*p* = 0.154, r = 0.08, R^2^ = 0.007	*p* = 0.230, r = 0.10, R^2^ = 0.009
hospital days	*p* < 0.0001, r = 0.41, R^2^= 0.167	*p* = 0.473, r = 0.04, R^2^ = 0.001	*p* = 0.01, r = 0.25, R^2^ = 0.063	*p* = 0.529, r = 0.04, R^2^ = 0.001	*p* = 0.83, r = 0.01, R^2^ = 0.0001		*p* = 0.85, r = 0.01, R^2^ = 0.0001	*p* = 0.01, r = 0.25, R^2^ = 0.063	*p* = 0.071, r = 0.11, R^2^ = 0.011	*p* = 0.488, r = 0.04, R^2^ = 0.001	*p* = 0.389, r = 0.07, R^2^ = 0.004
leukocyte	*p* = 0.765, r = 0.02, R^2^ = 0.0003	*p* = 0.337, r = 0.06, R^2^ = 0.003	*p* = 0.887, r = 0.01, R^2^ = 0.00007	*p* = 0.500, r = 0.04, R^2^ = 0.001	*p* = 0.62, r = 0.03, R^2^ = 0.0001	*p* = 0.85, r = 0.01, R^2^ = 0.0001		*p* = 0.041, r = 0.12, R^2^ = 0.01	*p* = 0.249, r = 0.07, R^2^ = 0.004	*p* = 0.650, r = 0.03, R^2^ = 0.0007	*p* = 0.764, r = 0.02, R^2^ = 0.0005
CRP	*p* = 0.072, r = 0.11, R^2^ = 0.01	*p* = 0.408, r = 0.05, R^2^ = 0.002	*p* = 0.014, r = 0.14, R^2^ = 0.02	*p* = 0.940, r = 0.00, R^2^ = 0.00001	*p* = 0.05, r = 0.12, R^2^ = 0.015	*p* = 0.01, r = 0.25, R^2^ = 0.063	*p* = 0.041, r = 0.12, R^2^ = 0.01		*p* = 0.879, r = 0.01, R^2^ = 0.00008	*p* = 0.642, r = 0.03, R^2^ = 0.0007	*p* = 0.966, r = 0.00, R^2^ = 0.00001
antibiotics	*p* = 0.490, r = 0.04, R^2^ = 0.001	*p* = 0.272, r = 0.06, R^2^ = 0.004	*p* = 0.151, r = 0.08, R^2^ = 0.007	*p* < 0.001, r = 0.21, R^2^ = 0.004	*p* = 0.314, r = 0.06, R^2^ = 0.003	*p* = 0.071, r = 0.11, R^2^ = 0.011	*p* = 0.249, r = 0.07, R^2^ = 0.004	*p* = 0.879, r = 0.01, R^2^ = 0.00008		*p* = 0.002, r = 0.18, R^2^= 0.003	*p* = 0.168, r = 0.11, R^2^ = 0.012
comorbidities	*p* = 0.233, r = 0.07, R^2^ = 0.004	*p* = 0.297, r = 0.06, R^2^ = 0.003	*p* = 0.441, r = 0.05, R^2^ = 0.002	*p* = 0.597, r = 0.03, R^2^ = 0.0009	*p* = 0.154, r = 0.08, R^2^ = 0.007	*p* = 0.488, r = 0.04, R^2^ = 0.001	*p* = 0.650, r = 0.03, R^2^ = 0.0007	*p* = 0.642, r = 0.03, R^2^ = 0.0007	*p* = 0.002, r = 0.18, R^2^ = 0.003		*p* = 0.013, r = 0.20, R^2^ = 0.03
type of intervention	*p* = 0.886, r = 0.01, R^2^ = 0.0001	*p* < 0.0001, r = 0.81, R^2^= 0.657	*p* = 0.554, r = 0.05, R^2^ = 0.002	*p* = 0.004, r = 0.22, R^2^ = 0.051	*p* = 0.230, r = 0.10, R^2^ = 0.009	*p* = 0.389, r = 0.07, R^2^ = 0.004	*p* = 0.764, r = 0.02, R^2^ = 0.0005	*p* = 0.966, r = 0.00, R^2^ = 0.00001	*p* = 0.168, r = 0.11, R^2^ = 0.012	*p* = 0.013, r = 0.20, R^2^ = 0.03	

## Data Availability

All data generated or analyzed during this study are included in this published article.
